# Effect of extended use N95 respirators and eye protection on personal protective equipment (PPE) utilization during SARS-CoV-2 outbreak in Singapore

**DOI:** 10.1186/s13756-020-00753-2

**Published:** 2020-06-15

**Authors:** Glorijoy Shi En Tan, Kyaw Zaw Linn, Margaret Mei Ling Soon, Shawn Vasoo, Monica Chan, Bee Fong Poh, Oon-Tek Ng, Brenda Sze-Peng Ang, Yee-Sin Leo, Kalisvar Marimuthu

**Affiliations:** 1 National Centre for Infectious Diseases, 16 Jalan Tan Tock Seng, Singapore, 308442 Singapore; 2 Tan Tock Seng Hospital, 11 Jalan Tan Tock Seng, Singapore, 308433 Singapore; 3 Lee Kong Chian School of Medicine, 11 Mandalay Road, Singapore, 308232 Singapore; 4 Yong Loo Lin School of Medicine, 10 Medical Drive, Singapore, 117597 Singapore

**Keywords:** SARS-CoV-2, Personal protective equipment, Extended use N95 respirator

**Dear Editor**

The ongoing Severe Acute Respiratory Syndrome Coronavirus (SARS-CoV-2) pandemic has resulted in shortage of personal protective equipment (PPE) worldwide [1]. The first positive case in Singapore was reported on 23 January 2020 when the outbreak began [2]. As the numbers of suspect and confirmed SARS-CoV-2 cases in Singapore rose, the largest proportion of them were cared for at the National Centre for Infectious Diseases (NCID), a purpose-built facility designed to strengthen Singapore’s capabilities in infectious disease management. We describe rates of PPE utilization during the outbreak and the impact of practicing extended use N95 respirators [3] and eye protection on their usage in outbreak wards.

We conducted the study between 1 February and 2 March 2020. All suspect and confirmed cases of SARS-CoV-2 were admitted to a single isolation room (12 air exchanges per hour). We included all patients admitted to both the general ward and intensive care unit. Healthcare workers (HCW) involved in their care were required to don full PPE comprising N95 respirator, eye protection, full sleeve gown and surgical gloves. HCW were advised to cluster clinical activities (for example blood taking, physical examination), and use ViSi Mobile (ViSi®), a wearable remote vital signs monitor, for routine monitoring to minimize entry into patient rooms.

Prior to 4 February 2020, N95 respirators and eye protection were single-patient use. On 4 February, reusable goggles were issued to each HCW. On 8 February, a directive was issued recommending extended use of N95 respirators and eye protection for repeated encounters with different patients, without changing between patients unless visibly contaminated or dislodged (Supplementary Appendix Floor Plan). Gowns and gloves remained as single-patient use.

During study period, a daily ward-level stocktake calculating the difference in balance PPE compared to preceding day, accounting for supplies replenished, was defined as utilization rate per day. A daily ward census was recorded. We applied linear regression using STATA 15.0 to compare change in PPE utilization per 100 patient-days (β) before and after extended use.

A total of 77 confirmed and 725 suspect cases were admitted during study period. Intensive care unit (ICU) admissions contributed to 199 (8.1%) patient-days (Fig. 1). The average utilization rate of single-use eye and N95 respirators per 100 patient-days reduced as a result of extended use policy. The average utilization rate of single-use eye protection and N95 respirators reduced from 1950 to 250 and 2490 to 1710 respectively after implementation.

**Fig. 1:**
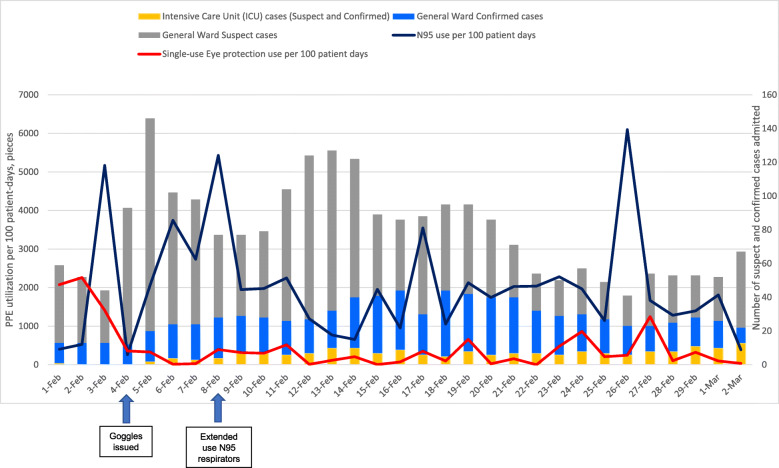
Utilization of PPE per 100 inpatient days during SARS-CoV-2 Outbreak in NCID

Before extended usage, the N95 respirator utilization per 100 patient-days steadily increased from 400 on 1 February to 5428.6 on 8 February (β = 521.22) (Supplementary Appendix Fig. A). After implementation, the N95 utilization rate dropped to 388.1 on 2 March (β = 11.04). Single–use eye protection utilization rate per 100 patient-days was 2076.3 on 1 February and had decreased to 1411.4 on 3 February (β = − 332.45) (Supplementary Appendix Fig. B). By 2 March, it decreased sharply to 32.84 (β = 4.84).

We describe the relative reduction in PPE utilization with extended eye protection and N95 respirator use. As of 23 March 2020, no HCW in our institution has been confirmed to have nosocomially acquired SARS-CoV-2 through staff surveillance and testing of symptomatic staff, reaffirming safety. In a preliminary analysis, N95 respirators and goggles of HCW were found not to be contaminated after patient contact [4, 5].

There are some limitations in our analysis. Data pertaining to PPE utilization were based on crude estimation of balance ward supplies counted on a daily basis and may not reflect exact utilization rate. Secondly, the implementation of extended N95 respirator use was recommended as of 8 February but not strictly enforced and cannot be assumed to be at 100% compliance. HCW were at liberty to change their N95 respirators when clinically indicated, if adjustments were required or if PPE was visibly soiled. Evaluation of compliance to PPE use are being planned for. Lastly, as this was a purely descriptive study, there may have been other factors confounding factors affecting PPE utilization rate that were not analysed.

As the SARS-CoV-2 pandemic continues to develop with cases anticipated to increase globally, there is ongoing need to review PPE stockpiles and rationalize PPE use. Intervening measures such as extended use of N95 respirators can safely help to reduce rapid consumption of limited supplies. Further modelling data on the utilization of PPE using different hospital admission strategies and PPE use are required in order to determine whether stockpiles of PPE will be sufficient in the medium to long term.

## Supplementary information

**Additional file 1:** Utilization rates of N95 respirator and single-use eye protection per 100 patient-days before and after extended use; Floor plan of isolation room.
