# Supplementary material for: Effect of extended use N95 respirators and eye protection on personal protective equipment (PPE) utilization during SARS-CoV-2 outbreak in Singapore
Source: Antimicrob Resist Infect Control. 2020 Jun 15;9:86. doi: 10.1186/s13756-020-00753-2 (PMC7294768; doi:10.1186/s13756-020-00753-2)
Supplement: Supplementary file 1 — Additional file 1: Utilization rates of N95 respirator and single-use eye protection per 100 patient-days before and after extended use; Floor plan of isolation room. [file 13756_2020_753_MOESM1_ESM.docx]

**Supplementary Appendix**

Figure A. Utilization Rate of N95 respirator use per 100 patient-days before and after extended use

Figure B. Utilization Rate of single-use eye protection per 100 patient-days before and after extended use

Floor plan - (Single bed with anteroom) illustrating how HCW don and doff PPE for extended eye protection and N95 respirators


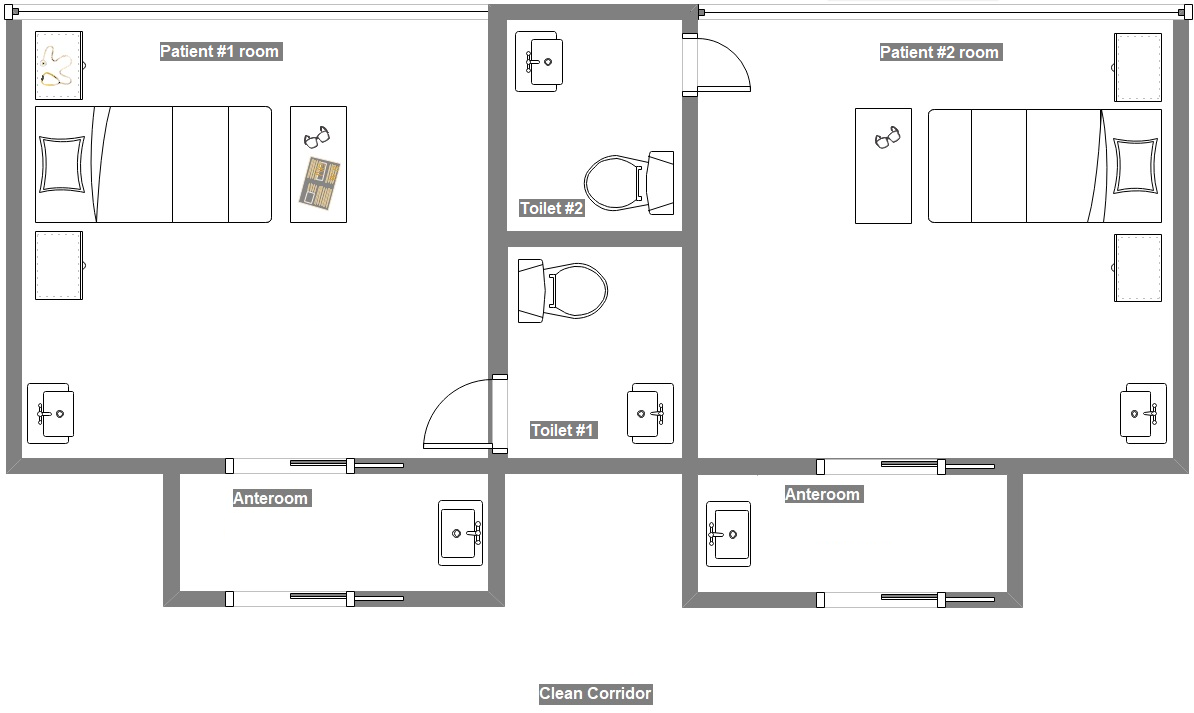


1

2

3

4

5

6

7

1 – Clean corridor - HCW dons N95 respirator and goggles

2- Anteroom - HCW dons shower cap, gown and gloves

3 – Patient’s room – after patient contact, HCW removes shower cap, gown and gloves

4 – HCW keeps on extended use N95 respiratory and goggles to enter anteroom of next patient

5 - Anteroom - HCW dons shower cap, gown and gloves

6 - Patient’s room – after patient contact, HCW removes shower cap, gown and gloves

7 – Anteroom – if no further patient contact is anticipated, HCW removes goggles (to wipe down) and extended use N95 respirator (to be discarded).
